# Load-Deflection and Friction Properties of PEEK Wires as Alternative Orthodontic Wires

**DOI:** 10.3390/ma10080914

**Published:** 2017-08-09

**Authors:** Yoshifumi Tada, Tohru Hayakawa, Yoshiki Nakamura

**Affiliations:** 1 Department of Orthodontics, Tsurumi University School of Dental Medicine, Yokohama 230-8501, Japan; nakamura-ys@tsurumi-u.ac.jp; 2 Department of Dental Engineering, Tsurumi University School of Dental Medicine, Yokohama 230-8501, Japan; hayakawa-t@tsurumi-u.ac.jp

**Keywords:** orthodontic wire, PEEK, static friction, load deflection, three-point bending

## Abstract

Polyetheretherketone (PEEK) is now attracting attention as an alternative to metal alloys in the dental field. In the present study, we evaluated the load-deflection characteristics of PEEK wires in addition to their frictional properties. Three types of PEEK wires are used: two sizes of rectangular shape, 0.016 × 0.022 in^2^ and 0.019 × 0.025 in^2^ (19-25PEEK), and rounded shape, diameter 0.016 in (16PEEK). As a control, Ni-Ti orthodontic wire, diameter 0.016 in, was used. The three-point bending properties were evaluated in a modified three-point bending system for orthodontics. The static friction between the orthodontic wire and the bracket was also measured. The load-deflection curves were similar among Ni-Ti and PEEK wires, except for 16PEEK with slot-lid ligation. The bending force of 19-25PEEK wire was comparable with that of Ni-Ti wire. 19-25PEEK showed the highest load at the deflection of 1500 μm (*p* < 0.05) in the case of slot-lid ligation. No significant differences were seen in the permanent deformation between Ni-Ti and all three PEEK wires (*p* > 0.05). No significant difference was seen in static friction between all three PEEK wires and Ni-Ti wire (*p* > 0.05). It is suggested that 19-25PEEK will be applicable for orthodontic treatment with the use of slot-lid ligation.

## 1. Introduction

Metal alloys such as stainless steel, cobalt-chromium (Co-Cr), and nickel-titanium (Ni-Ti) alloy are widely used as orthodontic wires. The elastic properties of metal alloys play a significant role in efficient tooth movement in orthodontic treatment [1]. Among them, Ni-Ti alloy is known to have a superelastic property [2,3]. The superelastic behavior means that an unloaded Ni-Ti alloy returns to its original shape after deformation. Numerous studies have reported on the elastic properties of Ni-Ti alloy. For example, Ni-Ti alloy has greater strength and a lower modulus of elasticity compared to stainless steel [4]. It is also reported that Ni-Ti alloy wire shows less permanent deformation and excellent springback qualities in comparison to stainless steel and Co-Cr wires [5].

However, metal alloy arch wires have the disadvantages of esthetics and metal allergies, so alternatives to metal alloys are needed in orthodontic treatment. Polymer materials have an advantage in esthetics. Some polymers are known to have higher mechanical strengths and are applied in industrial fields. Among them, polyetheretherketone (PEEK) is now attracting attention as an alternative in medical and dental fields. PEEK has a white color and excellent mechanical properties, as well as being known to be bio-inert [6]. Therefore, PEEK has been proposed for prosthodontics applications such as fixed prostheses and removal prostheses [7,8,9,10].

Maekawa et al. [11] evaluated the bending properties and water adsorption of PEEK to assess its feasibility as orthodontic wire. They compared some properties of PEEK with metal alloys and other polymers and concluded that PEEK has many advantageous properties and is a suitable candidate for esthetic metal-free orthodontic wire. However, they used PEEK plate that was a 1.0 × 1.0 mm square, which is not a suitable size for orthodontic wire. Orthodontic wire is normally a rectangular or rounded shape of 0.40–0.65 mm width or diameter. The size of wire influences the bending properties. In addition, they evaluated the three-point bending properties without any kind of ligature.

Kasuya et al. [12] investigated the effect of the difference of ligation on the load-deflection characteristics of Ni-Ti orthodontic wire. Three different ligation methods, namely, stainless steel ligature, slot-lid ligature (SL), and elastomeric ligature (EL), were employed, as well as no ligation (NL). Load-deflection curves were obtained by using the modified three-point bending system for orthodontics. They found that the load-deflection characteristics of Ni-Ti orthodontic wire were influenced by the kind of ligature. SL did not restrain the superelastic properties of Ni-Ti, but EL could restrain the superelasticity of Ni-Ti wire.

In addition to the load-deflection characteristics, the static friction between orthodontic brackets and wire is an important factor that influences orthodontic tooth movement. Less friction can facilitate tooth movement due to the efficient transmission of orthodontic force to the teeth and the orthodontic treatment period may be shortened without unfavorable anchorage loss or patient pain [13,14,15,16].

In the present study, we evaluated the load-deflection characteristics of PEEK wires of orthodontic size and rectangular and rounded shapes. The frictional properties were also evaluated. Ni-Ti wire was used as a control. The null hypothesis is that PEEK wire is applicable as an orthodontic appliance.

## 2. Results

### 2.1. Three-Point Bending Tests

**Figure 1:**
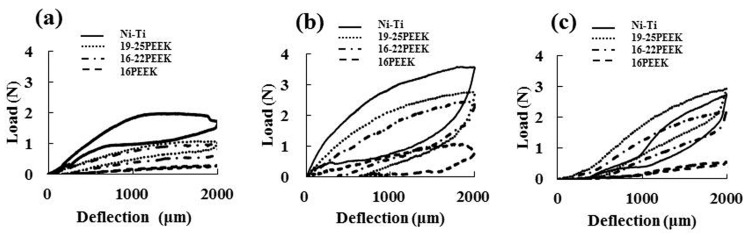
Load-deflection curves by three-point bending test (**a**) no-ligation; (**b**) elastomeric ligature; (**c**) slot lid.

Figure 1 shows the load-deflection curves of the three different PEEKs and Ni-Ti wires with different ligation methods. For NL (Figure 1a), the Ni-Ti wire showed typical superelastic behavior with an unloading plateau range of approximately 1 N. Permanent deformation was not clearly observed and the bending load of the Ni-Ti wire was higher than that of the three PEEK wires (*p* < 0.05). The PEEK wires showed similar load-deflection curves to the Ni-Ti wire except for 16PEEK, which showed a lower bending load as compared to the other two PEEK wires. Slight permanent deformation was found for all three PEEK wires.

Higher bending loads were obtained for EL and SL. The load deflection curves were similar among the Ni-Ti and PEEK wires except for 16PEEK with EL and SL (Figure 1b,c). For EL (Figure 1b), the Ni-Ti wire also showed a higher bending load than that of all three PEEK wires, and 16PEEK showed a lower bending load as compared to the other two PEEK wires (*p* < 0.05). A slight amount of permanent deformation was observed for the Ni-Ti wire, and a greater degree of permanent deformation was observed for all three PEEK wires. SL showed different load-deflection curves as compared to EL (Figure 1c). The bending force of 19-25PEEK wire was comparable with that of Ni-Ti wire. 16PEEK showed a lower bending load among the three PEEK wires. The degree of permanent deformation was almost the same for Ni-Ti and all three PEEK wires.

**Figure 2:**
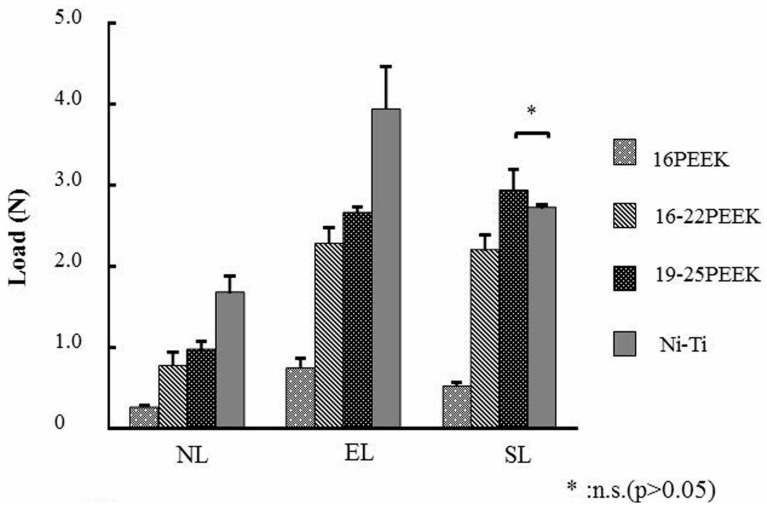
Load values of each wire at a maximum deflection of 2000 μm for the respective ligation methods.

**Figure 3:**
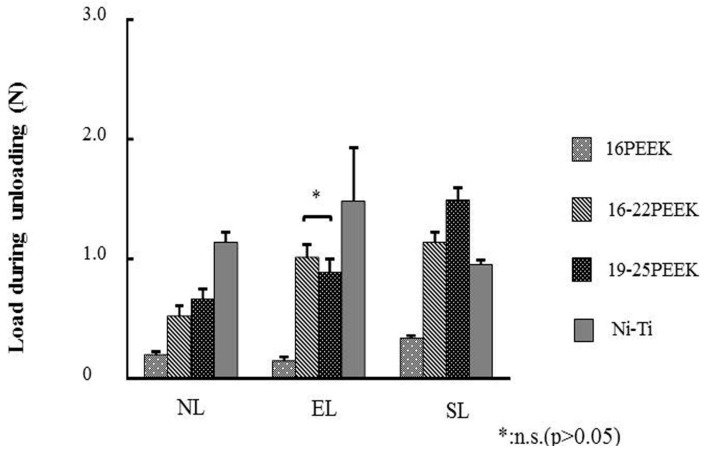
Load values of each wire at a deflection of 1500 μm during unloading for the respective ligation methods.

**Figure 4:**
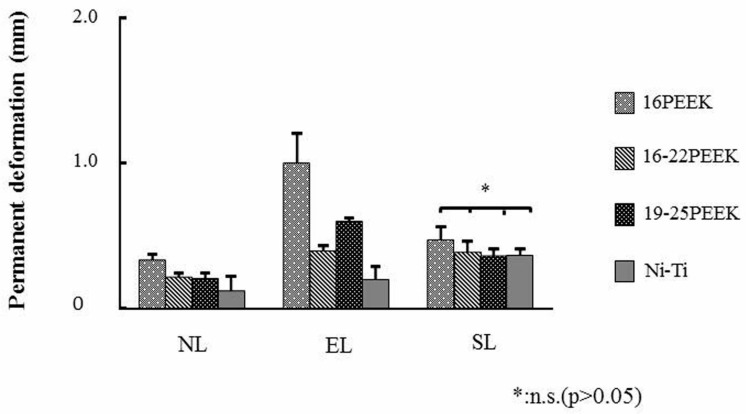
Apparent plastic deformation of each wire for the respective ligation methods.

Figure 2, Figure 3 and Figure 4 show the load at the maximum deflection of 2000 μm, the load at the deflection of 1500 μm during unloading, and the permanent deformation after three-bending tests with the different ligation methods respectively. For NL and EL, Ni-Ti wire showed a significantly higher load at the maximum deflection of 2000 μm than did the PEEK wires (*p* < 0.05), as shown in Figure 2. In contrast, no significant differences were seen in the load at the maximum deflection between Ni-Ti and 19-25PEEK for SL (*p* > 0.05). 16PEEK showed the lowest significant load for any of the three ligation methods (*p* < 0.05).

The load at the deflection of 1500 μm during unloading was evaluated (Figure 3). This load corresponds to the force for tooth movement in orthodontic treatment. Ni-Ti wire showed the highest load at the deflection of 1500 μm for NL and EL (*p* < 0.05).

For SL, loads at the deflection of 1500 μm of 16-22PEEK and 19-25PEEK wires were significantly higher than that of Ni-Ti wire (*p* < 0.05) and 19-25PEEK showed the highest load among the four wires at the deflection of 1500 μm (*p* < 0.05). 16PEEK also showed the lowest load deflection of 1500 μm for any of the three ligation methods (*p* < 0.05).

Permanent deformation was also influenced by the different ligation methods (Figure 4). Significant differences were observed in the permanent deformation among the four wires for NL and EL (*p* < 0.05), and the Ni-Ti wire showed the lowest significant permanent deformation (*p* < 0.05). However, no significant differences were observed in the permanent deformation between the Ni-Ti and three PEEK wires for SL (*p* > 0.05).

### 2.2. Stress Relaxation Tests

**Figure 5:**
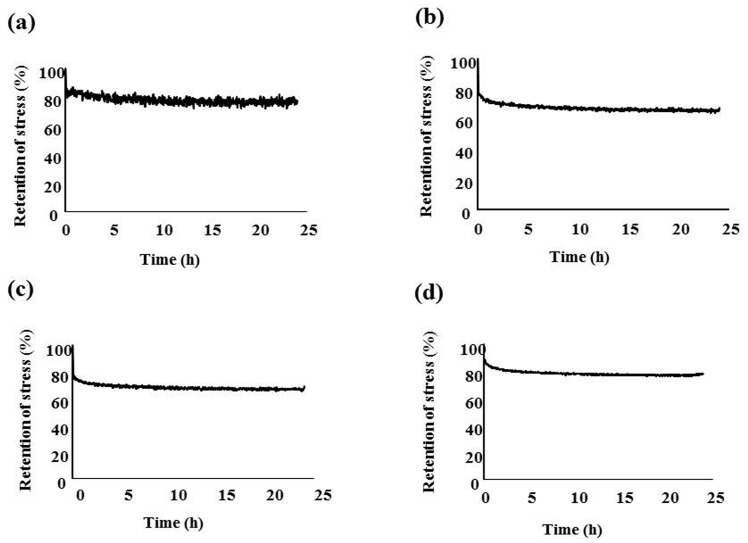
Rate of retention stress (**a**) rate of retention stress of 16 PEEK (Polyetheretherketone); (**b**) rate of retention stress of 16-22PEEK; (**c**) rate of retention stress of 19-25PEEK; (**d**) rate of retention stress of Ni-Ti.

Figure 5 shows the results of the stress relaxation tests. The pattern of stress relaxation was typical for a viscoelastic material. The rapid decrease of the load of the deflected wire was observed around 1.5–2 h and then the load became constant. The degree of retention of the initial force for 16PEEK and Ni-Ti was significantly higher than that for 16-22PEEK, as shown in Table 1 (*p* < 0.05).

### 2.3. Static Friction Tests

**Figure 6:**
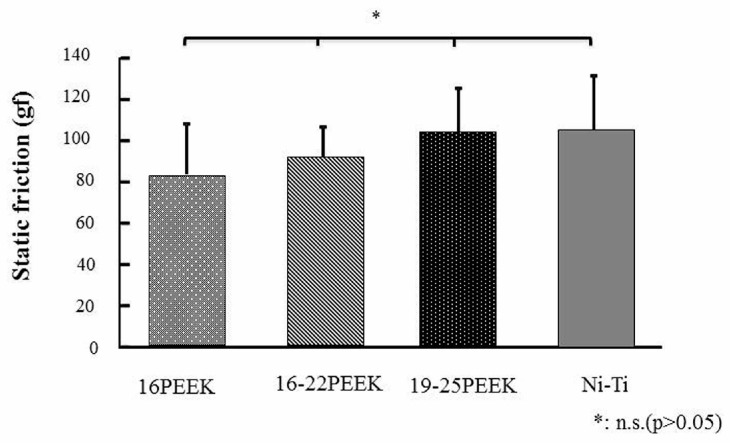
Static friction of each wire.

Figure 6 shows the static friction for each wire. No significant difference in static friction existed among all three PEEK wires and the Ni-Ti wire (*p* > 0.05).

**Figure 7:**
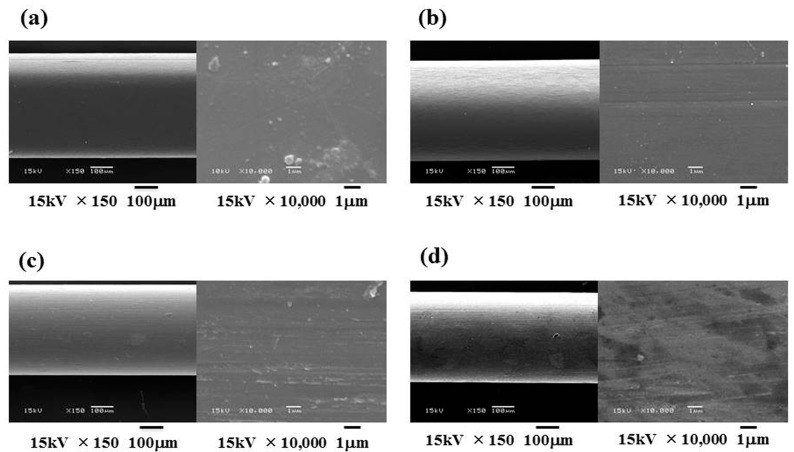
Scanning electron microscopy images of each wire before and after friction tests (**a**) surface of 19-25PEEK before friction test; (**b**) surface of 19-25PEEK after friction test; (**c**) surfaces of Ni-Ti before friction test; (**d**) surface of Ni-Ti after friction test.

SEM images of the 16PEEK and the Ni-Ti wires before and after friction tests are shown in Figure 7. No distinct differences in surface appearances for 16PEEK wire were observed before and after static friction tests. However, a rougher surface was identified for Ni-Ti wire after the friction test.

## 3. Discussion

In the present study, we evaluated the efficacy of PEEK wires as an alternative to Ni-Ti wire. Three-point bending tests were performed for simulating the early stage of orthodontic treatment, namely, leveling. Ni-Ti wire was used as a control. The load-deflection characteristics and the frictional properties of the PEEK wires were applicable as an orthodontic appliance. Especially, 19-25PEEK wire showed almost totally compatible properties with Ni-Ti wire. Thus, the null hypothesis is accepted.

PEEK is classified as super engineering plastic material which has high mechanical strength and chemically inert properties. Thus, PEEK could replace titanium, titanium alloy, Co-Cr-alloys and biological ceramics in orthopedic surgery [17]. For example, in medical and dental field, PEEK is applied as custom-made implants for craniofacial defects and dental implant under load bearing conditions [18,19].

Aside from esthetic problems, some shortcomings of metallic orthodontic appliances have been reported. For example, conventional metal orthodontic wire and brackets have been demonstrated to release nickel and chromium ions [20,21], and metal brackets couples with metal wires such as stainless steel or nickel-titanium (Ni-Ti) undergo galvanic reaction and corrosion in artificial saliva or in fluoride mouthwash [22]. Moreover, it has been reported that there is a substantial presence of nonmetallic inclusions, mainly in the form of silicates and oxides on the surface of Ni-Ti wires [23]. It has also been claimed that nonmetallic impurities already present at the postproductive stage and increased under influence of the oral environment must not be neglected. Thus application of the PEEK wire can greatly contribute to the development of metal-free devices in orthodontic treatment.

Ligation with stainless steel or elastomer is widely applied in orthodontic treatment. Stainless steel requires more skill in the ligation procedure and the ligation conditions are varied according to the clinician. Therefore we did not use stainless steel ligature.

Wires were directly held by elastomer in EL. The elasticity of elastomer is expected to give a continuous tightening pressure to the specimen wire in the bracket slot. SL ligation has been introduced for reducing the friction between wires and brackets [24]. For SL, the wire goes in the tunnel between the cover and the slot of the bracket. Thus, the load-deflection characteristics of the tested wires were influenced by the different ligation methods. Ni-Ti wire showed its superelastic properties only in the case of NL. No unloading plateau ranges for EL and SL were seen. Clear permanent deformation was observed for SL. Kasuya et al. [12] reported that the superelasticity of Ni-Ti was influenced by the differences of the ligation methods. In any event, evaluation of the load-deflection characteristics with ligation is necessary for clinical assessments of orthodontic wire. The use of no ligation does not reflect any clinical situation.

Load-deflection curves indicated that PEEK wires had similar elastic properties to Ni-Ti wire except for 16PEEK in EL. Thicker PEEK wire exhibited a higher load. The use of 16PEEK wire may be avoided for orthodontic use, because it had the lowest load among the tested wires.

Comparing the load at a maximum deflection of 2000 μm, 19-25PEEK showed a comparable load with Ni-Ti in SL ligation. The load at the maximum deflection of 2000 μm corresponded to the initial force when placing the wire in the orthodontic bracket at the first stage of orthodontic treatment. The distance of 2000 μm was simulated as the distance of a tooth before moving, according to previous papers [12,25]. The load at the deflection of 1500 μm during unloading was evaluated to represent the force of tooth movement. Proffit suggested that effective tooth movement needs a continuous optimum orthodontic force of 0.5–1.5 N [1]. 19-25PEEK showed a higher load than Ni-Ti in SL ligation. It is also suggested that tooth movement by 19-25PEEK might be more effective than that by Ni-Ti. Less permanent deformation is effective for tooth movement. The degree of permanent deformation of PEEK wires with SL ligation was similar to that of Ni-Ti.

Stress relaxation tests indicated that the load reduction of 16-22PEEK and 19-25PEEK was larger than that of Ni-Ti. However, 70–80% of the initial load was still maintained and the maintained value was sufficient for orthodontic treatment.

The static friction between PEEK wires and brackets was almost the same as that of Ni-Ti wire. For the friction test, EL was employed, in accordance with the previous report [26,27]. SEM revealed no damage of PEEK wires after friction, and the size and shape of PEEK did not influence the static friction. These results mean that tooth movement is not influenced by the size and shape of PEEK wires.

Based on the results of stress relaxation tests after 24 h, it is predicted that there will be no distinct difference in load-deflection behaviors over time between PEEK and Ni-Ti wire. For friction properties over time, frictional properties of PEEK will be maintained because of its high mechanical strength and chemically inert property. Detailed studies for load-deflection and friction properties over times will be needed as a next subject of our study.

In the oral conditions, bacterial adhesion and biofilm formation are crucial for dental materials. Hahnel et al. [28] reported that biofilm formation on a PEEK surface was equal or lower than that on a zirconia or titanium surface when used as an implant abutment. On the contrary, surface modification of PEEK with 2-methacryloyloxyethyl phosphorylcholine (MPC) has been reported to dramatically reduce the bacterial adhesion [29]. They concluded that hydration layer of MPC polymer influences the bacterial adhesion on the surface and also stated the difficulty for assessing the antibacterial efficiency of the material in physiological conditions. In vivo evaluation of antibacterial properties of PEEK wire should be further investigated.

Staining is another problem in the clinics. Heimer et al. [30] assessed the discoloration and stain removal potential on PEEK, polymethyl methacrylate and composite material after storage in different media and found that PEEK showed the significantly lowest color changes. It is suggested that PEEK wire will be a promising material regarding stain resistance.

Nonconventional ligature methoOKds such as self-ligation or nonconventional elastomers are now introduced to reduce the friction between wire and the bracket [31,32]. In these systems, wires go in tunnel like SL. Thus, similar load-deflection behaviors with SL will be predicted.

## 4. Materials and Methods

### 4.1. Materials

PEEK resin was extruded by using specially designed profile dies to create orthodontic wires with clinically relevant cross sections. Three types of PEEK wires were used: two sizes of rectangular shaped wires, 0.016 × 0.022 in^2^ (0.406 × 0.559 mm^2^) and 0.019 × 0.025 in^2^ (0.48 × 0.635 mm^2^), and one round shaped wire, diameter 0.016 in^2^ (0.406 mm^2^) (HOTTEY POLYMER., Tokyo, Japan). As a control, Ni-Ti orthodontic wire with a diameter of 0.016 in. (G&H Orthodontics, Franklin, IN, USA) was used. These wires are referred to as 16-22PEEK, 19-25PEEK, 16PEEK, and Ni-Ti, respectively.

As brackets, conventional plastic brackets (Clear Bracket, Dentsply Sirona, Tokyo, Japan) were used. The slot size was 0.022 × 0.028 in^2^ (0.559 × 0.711 mm^2^), the mesiodistal width was 2.9 mm, with no built-in torque or tip. The inside of the slot was covered with stainless steel.

### 4.2. Three-Point Bending Tests

**Figure 8:**
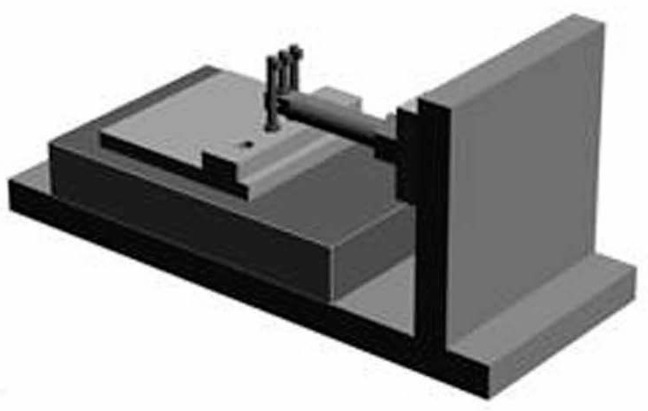
Three-point bending systems.

A modified three-point bending system for orthodontics [33] (Figure 8) was used for bending the orthodontic wires. This system was capable of digitally predetermining the bending speed and the amount of movement or deflection. In this system, the wire is bent between a rod connected to a load cell and two rods fixed in a linear gauge. The load is detected as strain by a compression load cell. This strain is converted to voltage by a dynamic strain amplifier and further converted from an analog to a digital signal by a sensor prior to the computer analysis.

**Figure 9:**
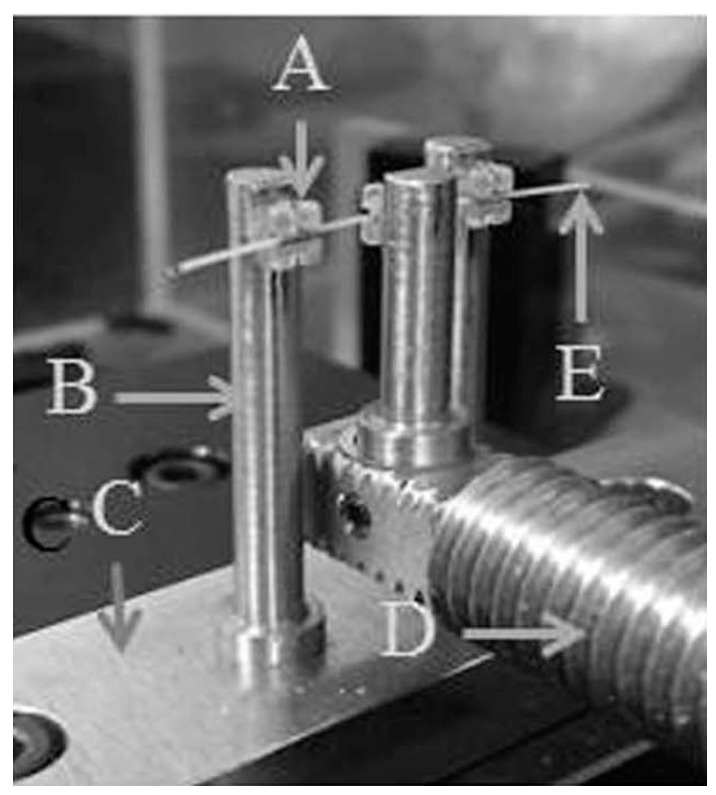
No ligation. A: bracket, B: rod, C: sensor linear gage, D: compression load cell, E: PEEK wire.

**Figure 10:**
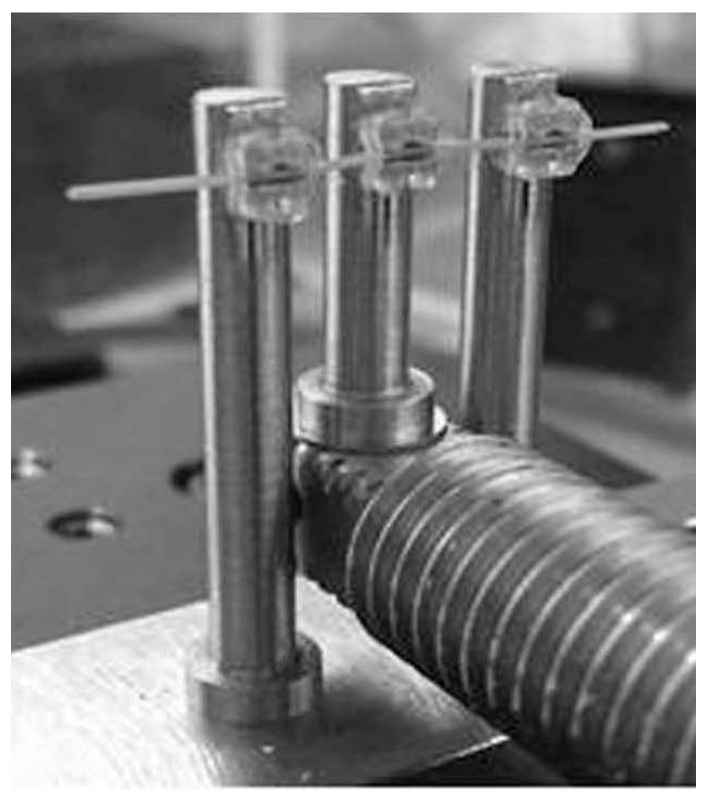
Elastomeric ligature.

**Figure 11:**
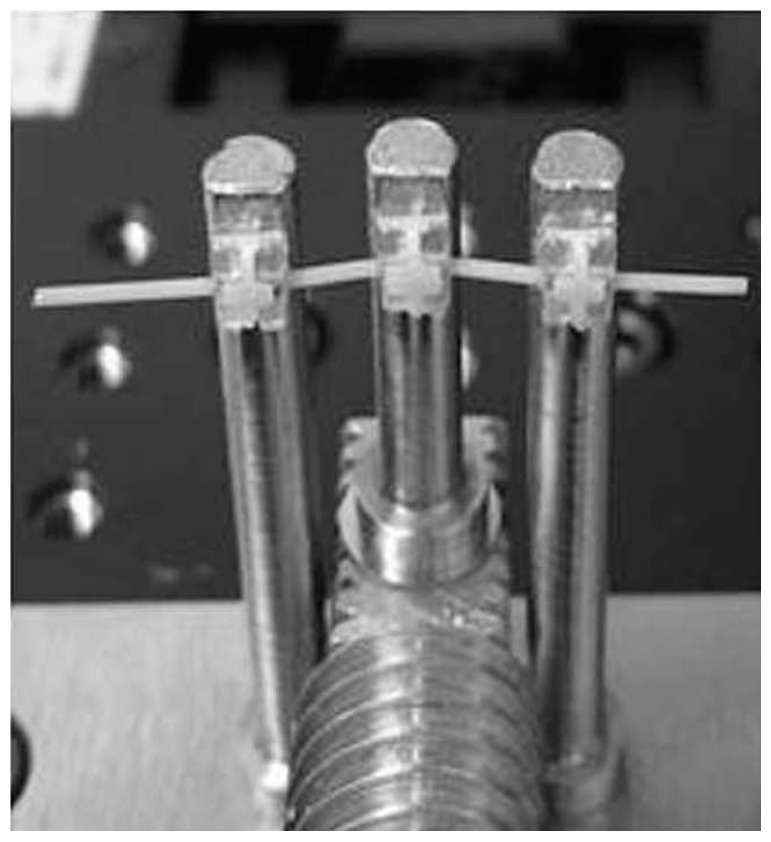
Slot lid ligature.

In the present study, the wire was inserted and ligated in the slots of three brackets, which were bonded to each of the three bender rods. This current three-point bending system is an improvement to a previous one [33]. The brackets could be positioned horizontally at the same position (Figure 9, Figure 10 and Figure 11). Three types of wire ligation methods, no ligation (NL), EL (module O, Tomy), and SL (Clear Snap, Dentsply Sirona), were evaluated. For NL, the wire was held with two outside rods and a center rod, as shown in Figure 9. For EL and SL, each bracket was bonded to each rod and facing in the same direction, as shown in Figure 10 and Figure 11. The initial distance between the neighboring bending rod and the center rod was set to 7 mm to represent an average inter-bracket distance in the lower anterior teeth. The bending cycle of loading and unloading was carried out at a speed of 10 μm/s. The maximum deflection was set to 2000 μm to reflect the clinical situation in which Ni-Ti arch wire is used to align mildly crowded anterior teeth.

For each ligation method, a comparison was made in the load-deflection curve between the maximum load at a deflection of 2000 μm, the load at a deflection of 1500 μm during unloading, and the maximum permanent deformation after unloading. Each combination of bracket and wire was measured five times. New material was used for each measurement.

Modified three-point bending system used in the present study. Bending at a maximum deflection of 2000 μm for three different methods.

### 4.3. Stress Relaxation Tests

Each wire was ligated in the brackets by SL, as described above, by using the modified three-point bending system. Then, the wire was deflected at 2000 μm and maintained at the same position at the deflection of 2000 μm for 24 h. The load decrease was monitored during the 24 h. After 24 h, the deflected wire was unloaded and the maximum permanent deformation was evaluated. Measurements for each wire were taken three times.

### 4.4. Static Friction Tests

**Figure 12:**
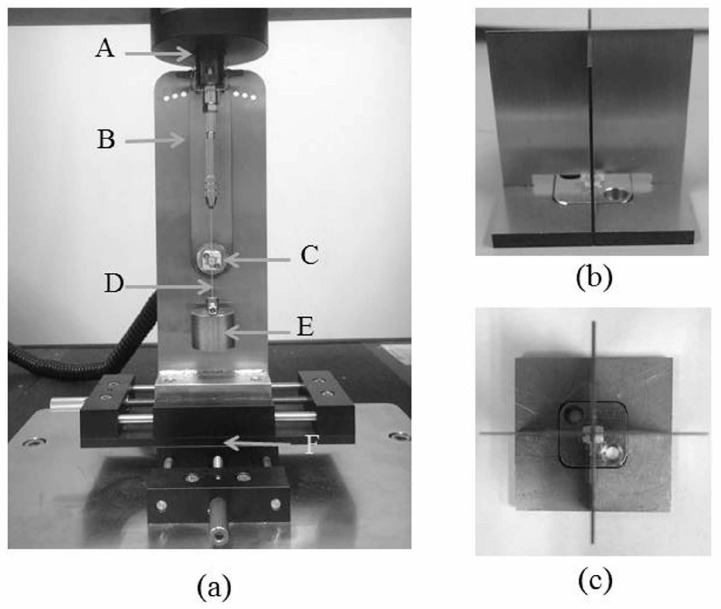
Friction testing systems (**a**) friction-testing device. A: load cell, B: base plate; C: bracket bonded stainless steel plate, D: wire, E: 150-g weight, and F: x-y table. (**b**,**c**) bracket-mounting device.

Measurements of the static friction between the orthodontic wire and the bracket were performed according to previous reports in the system shown in Figure 12 [26,27,34]. Each bracket was bonded accurately to the center of a stainless steel plate with unfilled adhesive resin (Superbond Orthomite, Sun Medical, Shiga, Japan) by using the bracket-mounting device shown in Figure 12b,c. The plate was then attached to the custom-made friction testing device, indicated by C in Figure 12a, and connected to a universal testing machine (Instron 5565, Instron Japan, Kanagawa, Japan). The adjustment plate was positioned by pushing a pin through the holes within that plate and the base plate B in Figure 12a.

The upper end of each 6 cm long wire was connected to the load cell of the universal testing machine and the lower end of the wire was connected to a 150-g weight. The wire was then ligated to the bracket by using EL ligation. Each bracket-wire combination was tested at angulations of 0°. Each wire was drawn through the bracket at a cross-head speed of 20 mm/min for a distance of 5 mm. The static friction between the bracket and the wire was measured as the peak force required to initiate movement of the wire through the bracket. This peak force was defined as the static friction. Measurements were carried out at room temperature and dry conditions. New material was used for each measurement. Each combination of bracket and wire was measured five times. New material was used for each measurement.

Before and after the friction tests, the surface conditions of 16PEEK and Ni-Ti were observed by a scanning electron microscope (SEM; JSM-5600LV, JEOL, Tokyo, Japan) at an accelerating voltage of 10 kV. Specimens were sputter-coated with Au prior to the SEM observations.

### 4.5 Statistical Analysis

For the statistical evaluation, the differences in load and deflection and in the static friction properties were tested by comparing the mean values among the four different wires with one-way ANOVA (*p* < 0.05, Windows Release 11.5.1, SPSS Inc., Chicago, IL, USA) and Tukey’s tests (*p* < 0.05). The evaluation of the differences in contact angles was performed by using the non-paired *t*-test (α = 0.05).

## 5. Conclusions

Within the limitations of the present study, it is suggested that 19-25PEEK will be applicable for orthodontic treatment with the use of SL ligation as an alternative to Ni-Ti wire. Evaluations of simulated clinical conditions including wet conditions should be further investigated, because eating, brushing or removing the food colorants and stains may influence the load-deflection characteristics and static friction of orthodontic wire in orthodontic treatment.

## Figures and Tables

**Table 1:** Rate of retention stress after 24 h. Values are given as mean (SD).

Wire	Rate of Retention Stress (%)
16PEEK	77.48 (1.13) ^a^
16-22PEEK	67.24 (5.20) ^a,b^
19-25PEEK	69.34 (4.92)
Ni-Ti	78.34 (0.53) ^b^

The same letters indicate statistically significant different rates (*p* < 0.05). ^a^ significant difference between 16PEEK and 16-22 PEEK. ^b^ significant difference between Ni-Ti and 16-22 PEEK.
